# Immunomodulation of Fucosyl-Lactose and Lacto-N-Fucopentaose on Mononuclear Cells from Multiple Sclerosis and Healthy Subjects

**Published:** 2006-06

**Authors:** S. Sotgiu, G. Arru, M. L. Fois, A. Sanna, M. Musumeci, G. Rosati, S. Musumeci

**Affiliations:** 1 *Institute of Clinical Neurology, University of Sassari, Sassari, Italy;*; 2 *Istituto Superiore di Sanita, Department of Hematology, Oncology and Molecular Medicine, Rome, Italy;*; 3 *Department of Pharmacology, Gynaecology and Obstetrics, Paediatrics, University of Sassari, Sassari, Italy, and Institute of Population Genetic, National Research Council (CNR), Alghero (SS), Italy*

**Keywords:** mononuclear cells, 2-fucosyl-lactose, lacto-N-fucopentaose I, multiple sclerosis

## Abstract

The 1,2-fucosyl-oligosaccharides, and among these the 2’-fucosyl-lactose (2’-FL) and lacto-N-fucopentaose (LNFP)-I, are quantitatively the most represented oligosaccharides of human milk. They are also seen to represent an important immune device to prevent nursing infants from severe infectious diarrhoea. Recent evidences show that the appearance of 2’-FL and LNFP-I in human colostrums is synchronised with the macrophage inhibition and that LNFP-III induces a Th2 response from the mouse peripheral immune system. Since mannosyl-fucosyl receptors are described on the macrophage surface, all these evidences allow us to investigate on the possible immune function of human 2’-FL and LNFP-I *in vitro* on LPS-activated mononuclear cells (MNC) from 12 patients with multiple sclerosis (MS) and 20 matched health controls (HC). We found that 2’-FL and LNFP-I significantly decrease, to a different extent, the MNC proliferation from both HC and MS patients, in a linear and dose-dependent manner. 2’-FL and LNFP-I also reduce the production of IL-12 and IFN-γ, particularly in MS patients as compared to HC (*p*=0.01 and *p*<0.001, respectively), while increasing that of IL-10. The overall immunomodulatory effect of 2’-FL and LNFP I here presented may represent a future therapeutic option for the abnormal immune response found in some monocyte-mediated diseases.

## INTRODUCTION

Oligosaccharides are important constituents of the human milk representing its third largest component (from 7.90 to 16.71 g/l) (1). Most human milk oligosaccharides are fucosylated, their production varies in relation to maternal Lewis blood group type and their distribution changes dynamically during the different phases of lactation, following an a priori program selected to the necessity of nursing infants (2). Among all fucosylated milk oligosaccharides, the α1,2-linked fucosylated glycans, which require the secretor gene for expression in human milk, are the dominant glycan structure found in the milk of secretor mothers, who represent the majority (approximately 80%) of mothers worldwide (3). Despite oligosaccharides were initially considered to be functionless, it has lately become apparent that at least some of them may protect infants against pathogens (2). The α1,2-linked fucosylated glycans are the first appearing, the longest-lasting and the most active in preventing infants from diarrhoea (4-6). *In vitro* and *in vivo* studies have demonstrated that α1,2-linked fucosylated oligosaccharides inhibit binding by Campylobacter, E. coli toxin and viruses to their target host cell receptors (5, 6) through a competitive mechanism.

It has also become progressively clear that 1,2-fucosylated milk oligosaccharides represent a component of an innate immune system by which the lactating mother protects her infant from environmental pathogens, particularly during the first months. Higher relative concentrations of 1,2-linked fucosylated oligosaccharides in human milk are associated with protection of breast-fed infants against diarrhoea of all infectious causes (3). Viruses, bacteria and toxins may become pathogenic after adhering to receptors located on the surface of intestinal epithelial cells (2, 7). Thus, intestinal cells are covered with 1,2 fucosylated glycoproteins and glycolipids (8, 9) produced by both epithelial cells and colonizing pathogens (10) which, through a host/pathogen protective molecular mimicry, prevent microrganisms from developing their full pathogenic potential (11-13).

A mannosyl-fucosyl receptor is described on the surface of macrophages (14). Perhaps not coincidentally, 2’-fucosyl-lactose (2’-FL) and lacto-N-fucopentaose (LNFP)-I, the first two appearing oligosaccharides in human colostrums (15), are synchronised with the concomitant macrophage deactivation, as demonstrated by the rapid decrease of colostrums chitotriosidase activity in the first three days of lactation (16). Interestingly, Okano and collaborators (17) have recently demonstrated the induction of a peripheral Th2 responses in mice by the immunisation with Schistosoma mansoni egg antigens, which was largely due to LNFP III, its predominant carbohydrate; the fucose residue of LNFP III was crucial for the immune response to rise. These result demonstrated that fucose-containing carbohydrates can show an ability to induce a Th2 response.

With this background in mind, we tested the effect of 2’-FL and of LNFP-I, both showing fucose residue in their extremities, on the proliferation and the cytokine production of E. coli lipopolysaccharide (LPS)-activated mononuclear cells from healthy subjects. As it cannot be a priori excluded that the potential immuno-modulatory effect of 2’-FL and LNFP I may be due to the presence of lactose, control experiments in parallel by using lactose alone were also performed.

Mononuclear cells from multiple sclerosis (MS) patients were also included in this study. The rational for choosing such cells is based on the evidence that MS is a chronic inflammatory disease of the central nervous system. Despite adaptive T- and B-cell antigen-specific responses are advocated at its patho-physiological basis, a growing body of data indicates that the innate immune response predominates in most MS lesion subtypes, which involves both resident and infiltrating macrophages as protagonists (18).

## MATERIALS AND METHODS

### Patients and controls

Twenty health controls (HC), 8 males and 12 females, mean age 27 ± 3) and 12 patients with definite MS (4 males and 8 females, mean age 28 ± 3 years, mean disease duration 6 ± 3 years) were recruited for this study. All patients and controls gave written consent to participate to this study.

### MNC cultures and oligosaccharides

According with worldwide adopted protocols, peripheral blood mononuclear cells (MNC) were obtained through a discontinuous density gradient as follows: heparinized blood was diluted 1:1 with PBS. Blood was layered on Ficoll (Lymphoprep; 1.077 g/ml; Nycomed, Oslo, Norway) at room temperature and centrifuged 30 min at 3000 rpm (no brake). Peripheral blood mononuclear cells were collected through a glass pipette and washed twice in cold PBS to avoid monocyte elimination due to their spontaneous plastic adherence. MNC were then counted. RPMI 1640 (Gibco, Paisley, Scotland) was used as culture medium, supplemented with 2 mM L-glutamine, 1% non-essential amino acids, 10% fetal calf serum, 50 U/ml penicillin and 50 μg/ml streptomycin (all from Gibco). The presence of monocytes within the MNC pool was confirmed on day 3 of culture by flow cytometry as positive for CD14 and HLA-DR, but lacking the markers for CD3 (T cells), CD19 and CD20 (B cells), CD16 and CD56 (NK cells), and CD123 (plasmocytoid DC). After 3 days the viability of the cultured cells was assessed through the use of the Turk’s reagent. More than 90% of cells were living at that time and available for the subsequent experiments.

**Figure 1:**
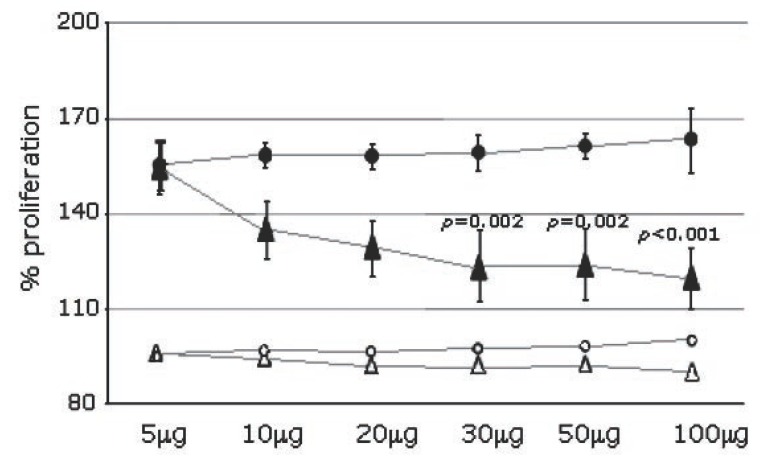
Dose-dependent and linear effect (± SD) of 2’-fucosyl-lactose (black triangles) and lactose (black circles) on LPS-driven MNC proliferation. On Y axis the proportion of the LPS-induced MNC proliferation (filled figures) compared to the unstimulated MNC (white figures). On X axis the concentration (μg/ml) of 2’-fucosyl-lactose and lactose in the MNC culture plates. The dose-dependent effect of 2’-fucosyl-lactose (white triangles) and lactose (white circles) on unstimulated MNC are also represented.

A preliminary experiment, represented on Figure 1, was designed to obtain the concentration of 2’FL to be added on LPS-activated MNC and to test the virtual inefficacy of a largely represented milk disaccharide such as lactose. For this preliminary test MNC from 6 HC were plated on a 96-well plate (200,000 MNC/well), either kept alone (3 wells/subject; white figures on Figure 1) or added with LPS, 10 μg/ml (filled figures on Figure 1). LPS-stimulated cells were either kept alone (3 wells/subject), added with 2’-FL in triplicate, at the physiological concentrations of 5, 10, 20, 30, 50 and 100 μg/ml (filled triangles) or pulsed with lactose, in triplicate, at the same concentrations as for 2’-FL (filled circles). With the significant response obtained, the 2’-FL concentration of 30μg/ml (0.06 mM) was used (See also Results section). Subsequently, 2’ FL and LNFP I (all by Sigma-Aldrich ns. ref. F 0393 and L 5908) were diluted in RPMI, both at an equimolecular concentration corresponding to 30g/ml 2’-FL (0.06mM) and used for the proliferation and cytokine tests.

### Proliferation assay

MNC from 20 HC and 12 MS were plated on a 96-well plate (200,000 MNC/well), either kept alone (3 wells/subject) or added in triplicate with 10 μg/ml LPS; then hydrocortisone (21,7 μg/ml; 0.06 mM) and equimolar amounts of 2’-FL (30 μg/ml), LNFP-I (51.2 μg/ml), and lactose (21.6 μg/ml), were added. A control experiment was made using placebo (i.e saline solution). After 48 hours, cell cultures were harvested, transferred in another 96-well microtiter plate and incubated with BrDU. After an additional 3 h culture, a proliferation assays was performed, in triplicate. Following instructions from the manufacturer (Amersham Biosciences) standard curves were performed for each assay. Optical density (OD) was measured using an enzyme-linked immunosorbent assays (ELISA) reader. According with the manufacturer indications, OD value of 1 is equivalent to 20 (3H)-thymidine cpm x 10^-3^ in a proliferation test of 24h duration and 500 L929 cell/well concentration.

### Cytokine production from MNC

On the proliferation test culture supernatants, technicians blind to clinical information performed the ELISA analysis for human IL-10, IL-12p40, TNF-α and IFN-γ (all from Euroclone, Switzerland). All assays for both standards and samples were performed in triplicate on 96-well plates, according with indications and suggestions from the manufacturers, and standard curves of the relationship between optic density and molecular concentration calculated accordingly. Biomarkers were expressed as pg/ml.

### Statistics

The results were reported as mean and standard deviation (SD). Differences between MNC proliferations in different conditions (expressed as optic density) were analysed by using a two-tailed paired and unpaired T-test. Significance level (p) was conventionally set at <0.05.

## RESULTS

### Proliferation assay in untreated and LPS-activated MNC

The mean proliferation of all untreated MNC was 3.6 ± 0.8 OD. When stratifying results according with health status, the mean proliferation was 3.4 ± 0.7 in MS patients and 3.8 ± 0.8 in HD (*p* not significant); no significant anti-proliferative effect was observed with the addition of either 2’-FL or lactose at either low or high concentrations (Figure 1, white triangles and circles). On the contrary, the mean proliferation of the LPS-stimulated MNC was 6.1 ± 2.4 OD in MS patients and 5.9 ± 1.2 in HC, which was significantly higher (157.9%, *p*=0.002) as compared to untreated cells but not significantly different between the two groups (data not shown).

### Effect of 2’FL and lactose on MNC proliferation (titration experiment, Figure 1)

2’-FL at the concentrations of 5, 10, 20, 30, 50 and 100 μg/ml inhibited LPS-driven MNC proliferation by, on average, 2.6% (*p* not significant), 15.6% (*p* not significant), 18.8% (*p* not significant), 23.4% (*p*=0.002), 22.7% (*p*=0.002) and 25.3% (*p*<0.001), respectively. This dose-dependent effect was linear. The 2’-FL concentration of 30 μg/ml (0.06 mM/L) was chosen for the remaining tests to be performed. On the contrary, lactose induced a slight and not significant increase of the LPS-driven MNC proliferation in this preliminary test, as shown on Figure 1. In details, lactose at the concentrations of 5, 10, 20, 30, 50 and 100 μg/ml increased the LPS-dependent MNC proliferation of 0.6, 2.6, 3.2, 3.9, 7.1 and 10.4% (*p* not significant for all concentrations), respectively. These results suggest that lactose has neither proliferative nor osmotic effects on MNC, since the MNC proliferation remained substantially unmodified while increasing the lactose concentrations.

### Overall effect of 2’-FL, LNFP-I, lactose and hydrocortisone on LPS-pulsed MNC in MS and HC subjects

**Figure 2:**
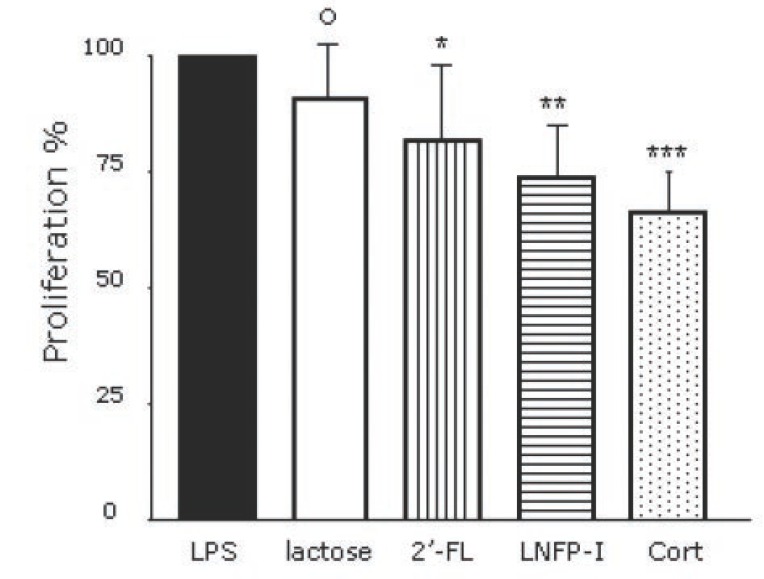
Effect of lactose, 2’-fucosyl-lactose (2’-FL), lacto-N-fucosyl-pentaose (LNFP)-I and hydrocortisone (cort) on the LPS-driven MNC proliferation. On Y axis the proportion of the MNC proliferation (100% is the level of the LPS-driven MNC proliferation). Lactose decrease LPS-driven MNC proliferation from 100 up to 91 ± 12% (*p*=not significant); 2’-FL up to 82 ± 16% (*p=0.04); LNFP-I up to 74 ± 11% (**p=0.002) and hydrocortisone (cort) up to 66.3 ± 8.7% (***p=0.0002); differences were also found between oligosaccharides (LNFP-I vs. 2’-FL *p*=0.039), between oligosaccharides (LNFP-I and 2’-FL) and lactose (°*p*=0.02 and 0,04 respectively) and between oligosaccharides and the steroid (hydrocortisone vs. LNFP-I: *p*=0.04; vs. 2’-FL: *p*=0.038).

When LPS-pulsed MNC from both MS and HC were added with lactose, 2’-FL, LNFP-I and hydrocortisone (at equimolecular amount of 0.06 mM) we observed a decrease of MNC proliferation (Figure 2). The effect of the add-on chemicals was expressed as percentage compared to LPS-treated MNC proliferation (assuming it as 100%; Figure 2).

Lactose induced a reduction of the LPS-driven proliferation of approximately 9% (from 100%, i.e. no effect, up to 91 ± 12%, i.e. 9% decrease; *p*=not significant); 2’-FL reduced the LPS-driven proliferation of 18 % on average (82 ± 16% of LPS-induced proliferation; *p*=0.04); LNFP-I decreased the LPS-driven proliferation of 26% on average (74 ± 11%: *p*=0.002) whilst hydrocortisone did it of approximately 34% (66.3 ± 8.7%; *p*=0.0002); significant differences were found between oligosaccharides themselves (LNFP-I vs. 2’-FL *p*=0.039), between oligosaccharides (LNFP-I and 2’-FL) and lactose (*p*=0.02 and 0,04 respectively) and, obviously, between oligosaccharides and steroid (hydrocortisone vs. LNFP-I: *p*=0.04; hydrocortisone vs. 2’-FL: *p*=0.038).

The effect of 2’-FL, LNFP-I, hydrocortisone and lactose was not significantly different when stratified according with health status (HC vs. MS) suggesting that 2’-FL, LNFP-I and hydrocortisone inhibit the MNC proliferative response to LPS independently from the MNC origin (data not shown).

### Measurement of soluble MNC products (details on Table 1 and Figure 3)

**Figure 3:**
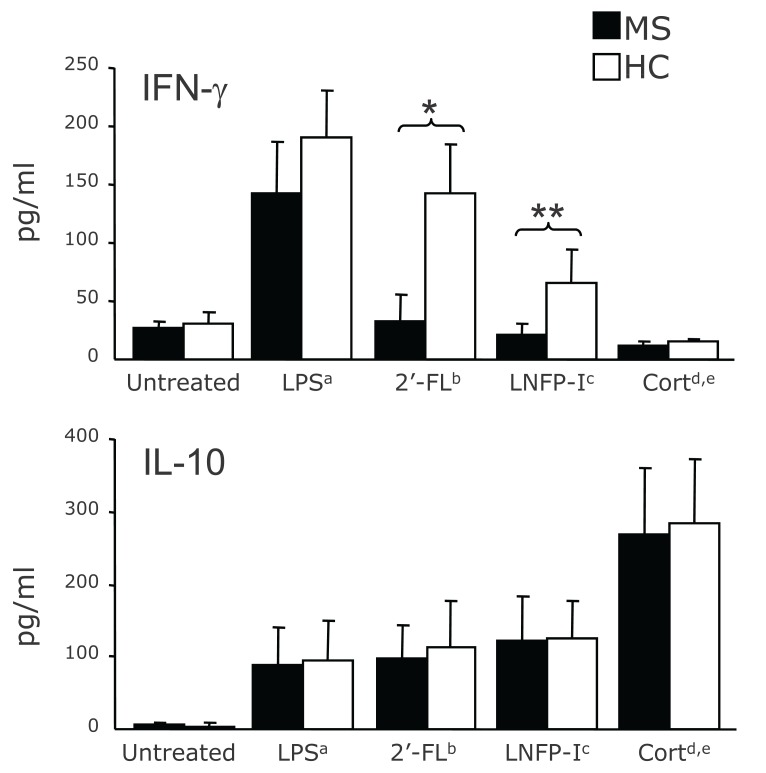
Effect of 2’-FL, LNFP-I and hydrocortisone on the level of IFN γ (upper graph) and IL-10 (lower graph) in supernatant of LPS-activated MNC from MS patients (black bars) and healthy controls (HC, white bars). Cytokine levels produced by untreated MNC are also reported. On the Y axis levels are expressed as pg/ml; *IFN-γ production in 2’-FLtreated MNC: MS vs. HC *p*<0.001; **IFN-γ production in LNFP-I-treated MNC: MS vs. HC *p*=0.01. a= LPS-treated vs. untreated MNC: *p*<0.01; b=2’FL- vs. LPS-treated MNC: *p*<0.05 for IFN-γ. *p*= not significant for IL-10; c=LNFP-Ivs. LPS-treated MNC: *p*<0.05; d=Hydrocortisone- vs. LPStreated MNC: *p*<0.01. e=Hydrocortisone- vs. 2’-FL and LNFP-I-treated MNC: *p*<0.05.

In untreated MNC, the baseline level of cytokine production was similar between MS and HC. Also, LPS-stimulated MNC from both HC and MS patients produced significantly higher levels of cytokines as compared to their untreated counterpart (*p*<0.01 for all cytokines), which did not significantly differ between the two groups. The treatment of the LPS-driven MNC with equimolecular dose of 2’-FL, LNFP-I and hydrocortisone 1μg/ml reduced the production of the cytokines TNF-α, IL-12p40 and IFN-γ, as indicated on Table 1, whilst the treatment with equimolar doses of lactose did not influence the overall cytokine secretion. The inhibitory effect of 2’-FL and LNFP-I on IL-12p40 and IFN-γ. production was particularly pronounced in the MS-derived MNC which significantly differed from the HC counterpart (*p*=0.01 for IL-12 and *p*<0.001 for IFN-γ after the use of 2’-FL; *p*=0.01 for both IL-12 and IFN-γ after the use of LNFP-I(see Table 1). IL-10 production was elevated after pulsing LPS-activated MNC with 2’FL, LNFP-I and hydrocortisone but did not significantly differ between MS and HC (see Table 1 and Figure 3).

**Table 1:** Production of cytokines from untreated and LPS-stimulated MNC of MS patients and health controls (HC). After pulsing with lactose, 2’-FL, LNFP-I and hydrocortisone, LPS-stimulated MNC decreased the TNF-α, IL-12, and IFN-γ production and increased the IL-10 production as compared to the unpulsed LPS-treated MNC. Following 2’-FL- and LNFP-I pulse the effect is significantly higher for IL-12 and IFN-γ from MS compared to the HC counterpart. All values are expressed as mean pg/ml ± standard deviation

	Untreated MNC		LPS (a)		Lactose		2’-FL (b)		LNFP-I (c)		Hydrocortisone (d,e)	
	MS	HC	MS	HC	MS	HC	MS	HC	MS	HC	MS	HC

TNF-α	120 ± 62	115 ± 62	351 ± 85	379 ± 75	324 ± 72	329 ± 68	268 ± 85	249 ± 75	248 ± 65	240 ± 75	155 ± 62	146 ± 52
IL-12p40	53 ± 12	49 ± 4	174 ± 115	123 ± 80	156 ± 80	119 ± 91	61 ± 11*	97 ± 40	55 ± 12*	92 ± 42	51 ± 10	46 ± 6
IFN-γ	26 ± 7	30 ± 11	143 ± 43	190 ± 40	135 ± 25	141 ± 66	32 ± 23**	142 ± 42	21 ± 9**	65 ± 30	12 ± 4	15 ± 3
IL-10	4.7 ± 5	3.9 ± 4	89 ± 52	96 ± 53	75 ± 46	90 ± 45	97 ± 48°	112 ± 64°	121 ± 62	126 ± 51	269 ± 92	285 ± 87

* IL-12 production: 2’-FL-treated MNC: MS vs. HC *p*=0.01; LNFP-I-treated MNC: MS vs. HC *p*<0.01.

** IFN- g production: 2’-FL-treated MNC: MS vs. HC *p*<0.001; LNFP-I-treated MNC: MS vs. HC *p*=0.01. a=LPS-treated vs. untreated MNC: *p*<0.01 for all cytokines; b=2’ FL- vs. LPS-treated MNC: *p*<0.05 for all cytokines with the exception of IL-10 (°*p*= not significant); c=LNFP-I- vs. LPS-treated MNC: *p*<0.05 for all cytokines; d=Hydrocortisone- vs. LPS-treated MNC: *p*<0.01 for all cytokines. e=Hydrocortisone- vs. 2’-FL and LNFP-I-treated MNC: *p*<0.05 for all cytokines.

## DISCUSSION

In our study we found clear evidences that the two milk oligosaccharides 2’-FL and LNFP-I, used in a concentration which could be considered physiological, negatively influenced MNC activation by decreasing their proliferation in a dose-dependent fashion; 2’-FL and LNFP-I also have an inhibitory role on IFN-γ and IL-12 production *in vitro*, particularly in MS patients, and a pro-releasing effect on the Th2 cytokine IL-10. Thus, these two oligosaccharides display an immunomodulatory effect, *in vitro*.

The results here described may reflect a cascade of events occurring within different MNC subpopulations (monocyte/macrophages, T-cell and others) which can be initiated by the 2’-FL and LNFP-I inhibitory effect on macrophages through the fucosyl receptor described on their surface (14). The two oligosaccharides induced a monocyte-macrophage down-regulation able of lowering the production of the macrophage-derived cytokines such as IL-12 and TNF. They might also indirectly reduce the macrophage-mediated T-cell proliferation and IFN-γ production. In order to shed some lights on this intriguing point, we have planned future studies on whether 2’-FL and LNFP-I may have inhibitory actions on purified populations of immune cells (e.g. T cells, B cells, monocytes).

We used the 2’ FL and LNFP-I 1,2-linked fucosyl-oligosaccharides compared to lactose for several reasons. 1,2-linked fucosyl-oligosaccharides are the first appearing oligosaccharides in colostrums in the first three days of lactation (15), quantitatively the most represented and the longest-lasting (2-6). Moreover, a role for the 1,2-fucosyl oligosaccharide LNFP III in inducing a Th2 switch in peripheral immune cells of mice is also documented(17). For all such reasons we omitted the use of oligosaccharides with other fucose residue linkage (1-4).

Besides being the main disaccharide contained in breast milk, lactose is a constitutive part of both 2’-fucosyllactose and lacto-N-fucopentaose-I. Virtually, we could not exclude a priori that the immunomodulatory effect seen for 2’-fucosyllactose and lacto-N-fucopentaose-I may be due to the presence of lactose. To test the specificity of the fucose-mediated effect, we have carried out parallel experiments by using lactose alone which turned out to induced minimal and not significant immune effects on LPS-treated MNC.

We are aware of the fact that other ligands of the mannose-fucose receptors are known to modulate the respiratory burst response of macrophages (19). Also, the different content of 2’ FL and LNFP-I in the milk reflects distinct activity of the fucosyl-transferase of the mothers and up to 10% Caucasian population lacks the 2’ fucosyl transferase (20).

In conclusion, we show for the first time that 2-FL and LNFP-I bear a novel immunomodulating function able to reduce the proliferation and detrimental cytokine production from peripheral lymphocytes through a macrophage-mediated inhibition. The specificity of this reaction, seems to be related to the characteristic structures of 2’FL and LNFP-I, both having the same terminal Fuc 1-2β Gal moiety. Comparing the model structures of these two oligosaccharides with Sweet, a program for constructing 3D models of saccharides (see website at http://www.dkfz.de/spec/sweet/doc/index.php), it appears that Fuc 1-2β Gal motive has the same conformation in each oligosaccharides.

Theoretical implications related to the present study are possible in the light of the current literature. The increase of cytokines such as IL-12 and IFN-γ play a determinant role in the complex cascade of events leading to the imbalance toward a detrimental Th1 environment in MS (21): IL-12 p40 mRNA level in unstimulated MNC increases in MS patients compared with controls (22) whilst MS patients experiencing a relapse have significantly increased IFN-γ production after mitogen-driven MNC stimulation compared to patients in remission, which is reduced after treatment with IFN-β (23). Furthermore, the therapeutical effect of IFN-β in MS is strictly associated to its enhancing effect on IL-10 (24).

Given their potential of opening a new avenue of therapeutic options in monocyte-mediated diseases, we think our findings warrant further evaluations.
